# What Is Needed to Eradicate Lymphatic Filariasis? A Model-Based Assessment on the Impact of Scaling Up Mass Drug Administration Programs

**DOI:** 10.1371/journal.pntd.0004147

**Published:** 2015-10-09

**Authors:** Randee J. Kastner, Christopher M. Stone, Peter Steinmann, Marcel Tanner, Fabrizio Tediosi

**Affiliations:** 1 Department of Epidemiology and Public Health, Swiss Tropical and Public Health Institute, Basel, Switzerland; 2 University of Basel, Basel, Switzerland; University of Minnesota, UNITED STATES

## Abstract

**Background:**

Lymphatic filariasis (LF) is a neglected tropical disease for which more than a billion people in 73 countries are thought to be at-risk. At a global level, the efforts against LF are designed as an elimination program. However, current efforts appear to aim for elimination in some but not all endemic areas. With the 2020 goal of elimination looming, we set out to develop plausible scale-up scenarios to reach global elimination and eradication. We predict the duration of mass drug administration (MDA) necessary to reach local elimination for a variety of transmission archetypes using an existing model of LF transmission, estimate the number of treatments required for each scenario, and consider implications of rapid scale-up.

**Methodology:**

We have defined four scenarios that differ in their geographic coverage and rate of scale-up. For each scenario, country-specific simulations and calculations were performed that took into account the pre-intervention transmission intensity, the different vector genera, drug regimen, achieved level of population coverage, previous progress toward elimination, and potential programmatic delays due to mapping, operations, and administration.

**Principal Findings:**

Our results indicate that eliminating LF by 2020 is unlikely. If MDA programs are drastically scaled up and expanded, the final round of MDA for LF eradication could be delivered in 2028 after 4,159 million treatments. However, if the current rate of scale-up is maintained, the final round of MDA to eradicate LF may not occur until 2050.

**Conclusions/Significance:**

Rapid scale-up of MDA will decrease the amount of time and treatments required to reach LF eradication. It may also propel the program towards success, as the risk of failure is likely to increase with extended program duration.

## Author Summary

Lymphatic filariasis (LF) is a disease caused by filarial worms transmitted by different types of mosquitos that can lead to massive disability, including elephantiasis and hydrocele. LF has no significant zoonotic reservoir and is thought to be a potentially eradicable disease through once yearly treatment distributed by mass drug administration (MDA). In this study, we set out to determine how many treatments and over how much time it might take to globally eliminate and eradicate LF under different levels of treatment intensities. We created a model that took into account country-specific and disease-specific variables, and found that if the current intensity of MDA is maintained, 3,409 million treatments distributed over the next 37 years will be required. However, if treatment is rapidly expanded to the entire at-risk population in all endemic countries, eradication could be achieved with 4,159 million treatments and in less than half the time. While our estimates suggest more time may be needed to reach LF elimination than what is currently projected, with continued commitment, eradicating LF is within reach.

## Introduction

Lymphatic filariasis (LF) is a neglected tropical disease (NTD) primarily prevalent in poor populations in 73 countries [1]. LF is caused by infection with *Wuchereria bancrofti*, *Brugia malayi*, or *B*. *timori* transmitted by a variety of mosquito genera [2]. Infection with the filarial nematodes can damage the lymphatic vessels, the main clinical manifestations being lymphedema, hydrocele, and elephantiasis [3]. In addition to disfigurement and disability, people affected by LF face stigma, social adversity, and economic hardship [4–6].

LF is spread by mosquitoes that take up circulating microfilarae (mf) in the peripheral blood of infected humans [7]. Administration of albendazole with ivermectin or diethylcarbamazine citrate (DEC) has been shown to reduce circulating mf to such low levels that transmission cannot be sustained [8]. For this reason, LF is one of six diseases considered to be potentially eradicable [9]. Accordingly, in 1997 the World Health Assembly (WHA) adopted resolution WHA 50.29, which calls for the elimination of LF as a public health problem and, in 2000, the World Health Organization (WHO) established the Global Program to Eliminate Lymphatic Filariasis (GPELF). The GPELF aims to eliminate LF in all endemic countries by 2020 through annual mass drug administration (MDA) maintained over multiple years [8]. The program benefits through donations from Merck & Co. and GlaxoSmithKline (GSK), which have pledged to provide enough ivermectin and albendazole, respectively, to achieve elimination, as well as from Eisai, which in 2010, pledged 2.2 billion DEC tablets [10, 11].

The GPELF has scaled up rapidly and is among the fastest growing disease elimination programs in the world [12]. By the end of 2013, 56 LF-endemic countries had carried out MDA, of which 15 are now undertaking post-MDA surveillance. In 2013 alone, more than 410 million anti-filarial treatments were distributed under the GPELF. However, the program is not without its challenges: mapping is incomplete in 12 countries, 14 countries requiring MDA are yet to begin, and many of the other endemic countries are targeting relatively small proportions of their at-risk populations [13]. Issues with compliance, contraindications of ivermectin and DEC in areas with hyper *Loa loa*-endemicity, and interruptions in funding also plague the program [14, 15]. At a global level, the efforts against LF could be considered a global elimination program (elimination of infection in some but not all countries) as the name suggests, or an eradication program (permanent reduction to zero of the worldwide incidence of infection) as implied by the stated aims of the program [13, 16, 17].

In order to assist decision makers in determining whether efforts for LF should be scaled up to try to achieve eradication, it has been proposed to use an analytic and deliberate methodology to produce evidence-based guidance on the rationale for investing [18, 19]. As part of this endeavor, we herein predict the duration of MDA necessary to reach local elimination for a variety of transmission archetypes using an existing model of LF transmission, outline plausible scale-up scenarios leading to global elimination and eradication, and estimate the number of treatments required under each scenario. Potential delays in implementation, previous progress, and different intensities of infection and transmission are also taken into account. Studies on the economic and financial costs, the impact on disease burden, and cost-effectiveness of these scenarios are to be published as companion papers.

## Methods

We have defined four hypothetical scenarios that differ in their geographic coverage and rate of scale-up. The global elimination scenario represents the case whereby countries continue with current practices. As such, it serves as the comparator against all other scenarios. The other three scenarios aim at reaching LF eradication through varying levels of MDA scale-up. Key assumptions and differences between the scenarios are outlined in Table 1. The number of years that each endemic country exceeded the minimum effective coverage rate of 65% in previous rounds of MDA, as well as the geographic coverage and rates of scale-up are provided in Table 2 (countries without previous rounds of MDA for LF) and Table 3 (countries that previously carried out MDA for LF). All scenarios were assumed to begin in 2014 and run until the final round of MDA has been distributed in each country under consideration. Though coverage rates above 65% are considered to be the lowest threshold necessary to be effective, the average programmatic coverage for countries that had previously achieved effective coverage was over 80%. Therefore, we presume that prospective MDA will continue to be performed at higher levels, and therefore assume MDA coverage to be fixed at 85%.

**Table 1:** Key features of the proposed scenarios for global elimination and eradication of LF.

	Global Elimination (comparator)	Eradication I	Eradication II	Eradication III
**Intervention**	MDA	MDA	MDA	MDA
**Coverage rate**	85%	85%	85%	85%
**Countries considered**	All LF endemic countries that have previously conducted MDA^¥^	All LF endemic countries^¥^, including all countries co-endemic with *L*. *loa*	All LF endemic countries^¥^, including all countries co-endemic with *L*. *loa*	All LF endemic countries^¥^, including all countries co-endemic with *L*. *loa*
**Rate of scale-up**	Countries with previous MDA continue at same rate as historically	Countries with previous MDA continue at same historical rate, countries without previous progress begin at an ‘average’ rate of MDA scale-up (schedule II)	Schedule I: All countries add 20% of their at-risk populations to the MDA schedule annually	All countries treat 100% of their at-risk populations annually

^¥^Assuming country requires MDA

**Table 2:** Countries without previous rounds of MDA for LF.

Country	Primary vector	Treatment^α^	At-risk population, 2012^¤^	Population growth rate, 2012^¥^	Scale-up schedule^±^	Delay^§^
Angola	*Anopheles*	IVM + ALB	12,090,000	3.1%	-/2/1/0	4
Brunei Darussalam	*Culex* *	DEC + ALB	15,000	1.4%	-/2/1/0	1
Chad	*Anopheles*	IVM + ALB	7,270,000	3.0%	-/2/1/0	4
Central African Republic	*Anopheles*	IVM + ALB	3,300,000	3.1%	-/2/1/0	4
Equatorial Guinea	*Anopheles*	IVM + ALB	420,000	2.8%	-/2/1/0	1
Eritrea	*Anopheles*	DEC + ALB	3,577,000	3.3%	-/2/1/0	4
Gabon	*Anopheles*	IVM + ALB	1,290,600	2.4%	-/2/1/0	1
Guinea	*Anopheles*	IVM + ALB	6,067,135	2.6%	-/2/1/0	1
New Caledonia	*Aedes*	DEC + ALB	12,378	1.6%	-/2/1/0	1
Palau	*Aedes*	DEC + ALB	20,044	0.7%	-/2/1/0	1
Republic of the Congo	*Anopheles*	IVM + ALB	2,600,000	2.6%	-/2/1/0	1
São Tomé and Príncipe	*Anopheles*	DEC + ALB	410,000	2.7%	-/2/1/0	1
South Sudan	*Anopheles*	IVM + ALB	1,659,558	4.3%	-/2/1/0	4
Sudan	*Anopheles*	IVM + ALB	19,893,779	2.1%	-/2/1/0	4
The Democratic Republic of Congo	*Anopheles*	IVM + ALB	49,140,000	2.7%	-/2/1/0	4
The Gambia	*Anopheles*	IVM + ALB	1,200,000	3.2%	-/2/1/0	1
Zambia	*Culex*	DEC + ALB	8,780,000	3.2%	-/2/1/0	4
Zimbabwe	*Culex*	DEC + ALB	6,000,000	2.7%	-/2/1/0	4

*Treatment durations for *Culex* spp. were used for countries in which primary vector species was unknown.

^**α**^Treatment assumed to occur once annually using diethylcarbamazine citrate (DEC) and albendazole (ALB), or in areas co-endemic with onchocerciasis, ivermectin (IVM) and albendazole (ALB)

^**¤**^ Preventive Chemotherapy Databank Lymphatic Filariasis [Internet]. WHO. 2015 [cited 2015 January 20]. Available from: http://www.who.int/neglected_diseases/preventive_chemotherapy/lf/en/.

^**¥**^ United Nations, Department of Economic and Social Affairs, Population Division (2013). World Population Prospects: The 2012 Revision, Key Findings and Advance Tables. Working Paper No. ESA/P/WP.227.

^±^ Refers to MDA schedules assumed to be used by these countries for the purposes of our analysis for the global elimination scenario, eradication I, eradication II, and eradication III scenarios, respectively. In schedule I, two deciles (20%) of the at-risk population are added to the MDA schedule annually. In schedule II, one decile is added annually. In schedule III, one decile is added every 2 years, and in schedule IV, one decile is added every 3rd year (see: Rate of Scale-Up and History of Control). ‘-‘ refers to a continued absence of an MDA program. ‘0’ refers to instantaneous scale-up.

^§^A 4-year delay was assumed for countries that have not completed LF mapping, while a 1-year delay was assumed for those that have completed mapping but have not previously carried out MDA.

**Table 3:** Countries that previously carried out MDA for LF.

Country	Primary vector	Treatment^α^	At-risk population, 2012^¤^	Population growth rate, 2012^¥^	Previous effective years^¤^	Scale-up schedule^±^
>50% targeted						
Burkina Faso	*Anopheles*	IVM + ALB	16,779,208	2.9%	11	1/1/1/0
Cameroon	*Anopheles*	IVM + ALB	17,091,469	2.5%	5	1/1/1/0
Côte d'Ivoire	*Anopheles*	IVM + ALB	14,000,000	2.3%	1	1/1/1/0
Comoros	*Culex*	DEC + ALB	514,110	2.4%	5	1/1/1/0
Egypt	*Culex*	DEC + ALB	536,443	1.7%	11	1/1/1/0
Fiji	*Aedes*	DEC + ALB	529,984	0.8%	7	1/1/1/0
French Polynesia	*Aedes*	DEC + ALB	274,544	1.1%	10	1/1/1/0
Ghana	*Anopheles*	IVM + ALB	11,925,399	2.2%	11	1/1/1/0
Haiti	*Culex*	DEC + ALB	10,732,356	1.4%	10	1/1/1/0
India	*Culex*	DEC + ALB	617,170,000	1.3%	15	1/1/1/0
Kenya	*Culex* *	DEC + ALB	3,421,741	2.7%	3	1/1/1/0
Lao PDR	*Culex* *	DEC + ALB	132,644	1.9%	2	1/1/1/0
Liberia	*Anopheles*	IVM + ALB	3,600,000	2.7%	0	1/1/1/0
Malawi	*Anopheles*	IVM + ALB	14,807,685	2.9%	5	1/1/1/0
Mali	*Anopheles*	IVM + ALB	16,166,882	3.0%	7	1/1/1/0
Mozambique	*Anopheles*	IVM + ALB	17,114,949	2.5%	3	1/1/1/0
Nepal	*Culex*	DEC + ALB	15,755,990	1.2%	10	1/1/1/0
Niger	*Anopheles*	IVM + ALB	12,467,592	3.8%	4	1/1/1/0
Philippines	*Aedes*	DEC + ALB	29,383,286	1.7%	9	1/1/1/0
Samoa	*Aedes*	DEC + ALB	186,649	0.8%	5	1/1/1/0
Sierra Leone	*Anopheles*	IVM + ALB	6,667,687	1.9%	5	1/1/1/0
Thailand	*Aedes*	DEC + ALB	73,495	0.3%	11	1/1/1/0
Tuvalu	*Aedes*	DEC + ALB	10,373	0.2%	4	1/1/1/0
Uganda	*Anopheles*	IVM + ALB	14,464,244	3.4%	5	1/1/1/0
*30–50% targeted*						
Dominican Republic	*Culex*	DEC + ALB	249,803	1.3%	6	2/2/1/0
Guyana	*Culex*	DEC + ALB	690,869	0.6%	2	2/2/1/0
Indonesia	*Culex*	DEC + ALB	113,283,453	1.2%	7	2/2/1/0
Myanmar	*Culex*	DEC + ALB	41,666,403	0.8%	9	2/2/1/0
Timor Leste	*Anopheles*	DEC + ALB	1,180,067	2.9%	3	2/2/1/0
United Republic of Tanzania	*Culex*	IVM + ALB	45,173,251	3.0%	11	2/2/1/0
*20–30% targeted*						
Bangladesh	*Culex*	DEC + ALB	77,230,000	1.2%	14	3/3/1/0
Benin	*Anopheles*	IVM + ALB	3,747,913	2.7%	11	3/3/1/0
Guinea Bissau	*Anopheles*	IVM + ALB	1,582,496	2.4%	1	3/3/1/0
Malaysia	*Anopheles*	DEC + ALB	1,266,123	1.7%	7	3/3/1/0
Nigeria	*Anopheles*	IVM + ALB	108,526,381	2.8%	5	3/3/1/0
*<20% targeted*						
Brazil	*Culex*	DEC	1,700,000	0.9%	4	4/4/1/0
Ethiopia	* *Culex*	IVM + ALB	30,000,000	2.6%	4	4/4/1/0
Kiribati	*Culex*	DEC + ALB	103,058	1.5%	5	4/4/1/0
Madagascar	*Anopheles*	DEC + ALB	18,602,379	2.8%	6	4/4/1/0
Micronesia	*Aedes*	DEC + ALB	11,241	0.1%	1	4/4/1/0
Papua New Guinea	*Anopheles*	DEC + ALB	5,602,188	2.2%	1	4/4/1/0
Senegal	*Anopheles*	IVM + ALB	5,314,600	2.9%	3	4/4/1/0

*Treatment durations for *Culex* spp. were used for countries in which primary vector species was unknown.

^**α**^Treatment assumed to occur once annually using diethylcarbamazine citrate (DEC) and albendazole (ALB), or in areas co-endemic with onchocerciasis, ivermectin (IVM) and albendazole (ALB)

^**¤**^ Preventive Chemotherapy Databank Lymphatic Filariasis [Internet]. WHO. 2015 [cited 2015 January 20]. Available from: http://www.who.int/neglected_diseases/preventive_chemotherapy/lf/en/.

^**¥**^ United Nations, Department of Economic and Social Affairs, Population Division (2013). World Population Prospects: The 2012 Revision, Key Findings and Advance Tables. Working Paper No. ESA/P/WP.227.

^±^ Refers to MDA schedules assumed to be used by these countries for the purposes of our analysis for the global elimination scenario, eradication I, eradication II, and eradication III scenarios, respectively. In schedule I, two deciles (20%) of the at-risk population are added to the MDA schedule annually. In schedule II, one decile is added annually. In schedule III, one decile is added every 2 years, and in schedule IV, one decile is added every 3rd year (see: Rate of Scale-Up and History of Control). ‘0’ refers to instantaneous scale-up.

### Scenario Development

Scenarios were developed by first reviewing the WHO preventive chemotherapy (PCT) databank to assess progress made towards LF elimination as of 2012 [13]. The scenarios were further refined, with key assumptions agreed upon in a series of technical advisory group meetings, including stakeholders from WHO, Centers for Disease Control and Prevention (CDC), funders, pharmaceutical companies, and program managers from endemic countries.

In the global elimination scenario, countries that have not yet started will not start, and countries that have started continue according to their assigned level of scale-up (see: *Rate of scale-up*). In the eradication I scenario, countries that have already started MDA continue as in the global elimination scenario and countries that have not yet started implement MDA following an ‘average’ level of scale-up. The eradication II scenario represents the case in which all countries scale-up MDA more quickly (fast). Eradication III serves as the ‘best case’ scenario, whereby all endemic countries provide MDA to their entire at-risk populations immediately. Thus, this analysis provides insight into the differences in the amount of time and treatments required to extend elimination efforts to all endemic countries (eradication I), increase MDA intensity (eradication II) and, most ideally, scale-up instantaneously (eradication III).

### Assumptions Regarding Interventions and Loiasis Co-endemicity

An important assumption underlying this study is that annual MDA using DEC with albendazole, or, in onchocerciasis-endemic countries, ivermectin and albendazole, will be sufficient to reduce circulating mf enough to interrupt the transmission cycle of LF if maintained for an appropriate number of years. Therefore, hardly predictable features that could undermine success, including systematic non-compliance with MDA, but particularly events such as civil unrest and humanitarian emergencies (e.g. earthquakes in Haiti and Nepal; Ebola epidemic in West Africa) that could compromise the health system’s capacity, could not be accounted for. We also assume that countries undertake MDA without interruption.

Administration of ivermectin to communities with high prevalence (>40%) of *L*. *loa* is contraindicated, as the microfilaracidal actions of the drug poses an unjustifiably high risk of causing severe adverse events. As such, the WHO provisionally recommends the LF program to instead treat these areas with albendazole monotherapy distributed bi-annually and vector control [20]. Here we assume that this strategy will be equally efficacious as annual albendazole-ivermectin, and thereby assume the number of years of MDA required in areas co-endemic with *L*. *loa* to be equivalent to the number of years required with albendazole-ivermectin.

### Rate of Scale-Up and History of Control

The GPELF advises LF endemic countries to conduct MDA for 4–6 years [8]. This duration only holds at a country level if all endemic areas are treated simultaneously. To incorporate scaling-up of geographic coverage for each scenario, we divided each country’s at-risk population into deciles, and assumed MDA to start in subsequent deciles after varying durations according to four schedules of scale-up. In schedule I (fast), 20% of the at-risk population is added to the MDA schedule annually. In schedule II (average), one decile is added each year, in schedule III (slow) one decile is added every two years and in schedule IV (very slow) this period is three years.

In the global elimination scenario, scale-up is based upon the proportion of the at-risk population each country previously targeted. In order to be allocated to schedule I, the at-risk population targeted in the most recent round of MDA had to exceed 50%. Schedule II has been assigned to countries previously targeting 30–50%, schedule III to those targeting 20–29.9%, and schedule IV to those targeting <20%. Rather than attempting to recreate the progress of each country exactly, we used these categories to incorporate a range of scale-up levels encountered. Previous progress made towards local elimination was further taken into account by counting the number of previously effective years of MDA, which was considered as any year in which program coverage within the targeted area (regardless of the at-risk population targeted) exceeded 65%. We then subtracted the number of effective years previously achieved from the number of years of MDA deemed necessary (see below: Transmission Archetypes; Table 4) in order to determine the number of years of MDA remaining.

**Table 4:** Estimates of the number of annual MDA rounds needed to reach local LF elimination by transmission archetypes, based on sets of 500 simulations using EpiFil and assuming 85% coverage.

Primary vector	Treatment^α^	Baseline MF prevalence			
		5%	10%	15%	20%
*Anopheles* spp.	DEC + ALB	6	6	7	7
	IVM + ALB	7	9	11	11
*Culex spp*.	DEC + ALB	9	10	11	11
	IVM + ALB	11	13	15	15

^**α**^Treatment assumed to occur once annually using diethylcarbamazine citrate (DEC) and albendazole (ALB), or in areas co-endemic with onchocerciasis, ivermectin (IVM) and albendazole (ALB)

The number of rounds corresponds to the minimum at which at least 97.5% of simulations went to elimination.

### Delays

For all scenarios, we assume that countries that have finished mapping but not begun MDA have a 1-year delay, whereas countries that have not completed mapping nor begun MDA have a 4-year delay. While countries face challenges of different magnitudes and require different durations to map, the 4-year delay assumed corresponds to the average number of years that mapping took in countries with available data to support the calculation [13].

### Prevalence Data

To account for heterogeneity in transmission intensity within countries, we obtained paired baseline circulating filarial antigenaemia prevalence, measured through immunochromatographic tests (ICTs), and mf prevalence data from sentinel site surveys from program countries across the AFRO region. As specified by the WHO, these surveys involve collecting fingertip blood, between 10 p.m. and 2 am. from at least 300 participants aged five years and above [21]. We gained additional access to ICT prevalence data from mapping studies in 17 African countries. The relationship between mf and antigenaemia prevalence was estimated using the non-parametric regression proposed by Passing and Bablock, which assumes linearity and uncertainties in both variables [22]. The regression equation calculated from the paired prevalence data was then used to infer mf prevalence from the ICT mapping data.

We determined the percentage of the at-risk population that fell into prevalence quartiles: <5%, 5–10%, 10.1–15%, >15%, for each country that provided district level prevalence data. To account for uncertainties in this approach, we took 500 random draws from a multinomial distribution with probabilities based on weighted averages from the dataset and assumed these to be the possible ranges of pre-intervention prevalence distributions for all countries in our analysis.

### Transmission Archetypes

It has been theoretically demonstrated that the required duration of MDA is region-specific and dependent on various factors, including drug regimen and level of coverage, vector species, and pre-intervention transmission intensity [23–25]. In order to broadly capture the heterogeneous transmission patterns of LF, we defined transmission archetypes (Table 4). In addition to prevalence levels and drug regimens, we accounted for differences in transmission between *Anopheles* spp. and *Culex* spp., which notably differ in their mf-density dependent likelihood of becoming infected [26]. Predicting regional anopheline- or culicine-mediated LF transmission has been shown to require different model formulations and parameterizations [27]. For our analysis we made several simplifications: we assumed transmission of *W*. *bancrofti* by *Aedes* spp. was similar to transmission efficacy by *Culex* spp., while transmission of *Brugia* spp. was assumed to be comparable to *W*. *bancrofti* transmission by *Anopheles* spp. Where the primary vector was unclear, infection by *Culex* spp. was assumed in order to avoid underestimating the number of MDA rounds required.

### Modeling the Number of MDA Rounds Required to Reach Local Elimination

The duration of MDA required to eliminate LF was predicted for the transmission archetypes using a deterministic model of LF transmission, EpiFil [28]. The model used for the current analysis has been described in detail, validated against multiple data sets for both transmission settings with *Anopheles* spp. and *Culex* spp., and used extensively to predict LF intervention outcomes [28–31]. Details on model structure, equations, and the approach to obtaining parameter estimates are provided in Supporting Text 1: LF model description.

For all transmission archetypes, we ran 500 simulations of once-yearly MDA of varying total durations, drawing from a range of parameter estimates. The lowest number of rounds at which the 95^th^ percentile range of the simulations resulted in an mf prevalence below 1% 50 years after the start of the MDA program was taken as a conservative measure of the number of rounds required to ensure elimination.

### Calculating the Number of Future Treatments Required

Population at-risk figures were taken from the WHO PCT database for 2012 and adjusted for population growth using country-specific 2012 United Nations estimates [13, 32]. MDA coverage rates were assumed to be 85% for all countries. Except for areas co-endemic with *L*. *loa*, treatments are assumed to occur annually. Based on the pre-intervention prevalence distributions, we developed 500 estimates of the number of treatments needed for each country and scenario. Results are reported as the mean number of treatments by region and scenario, along with 95% credible intervals (CI).

## Results

**Fig 1:**
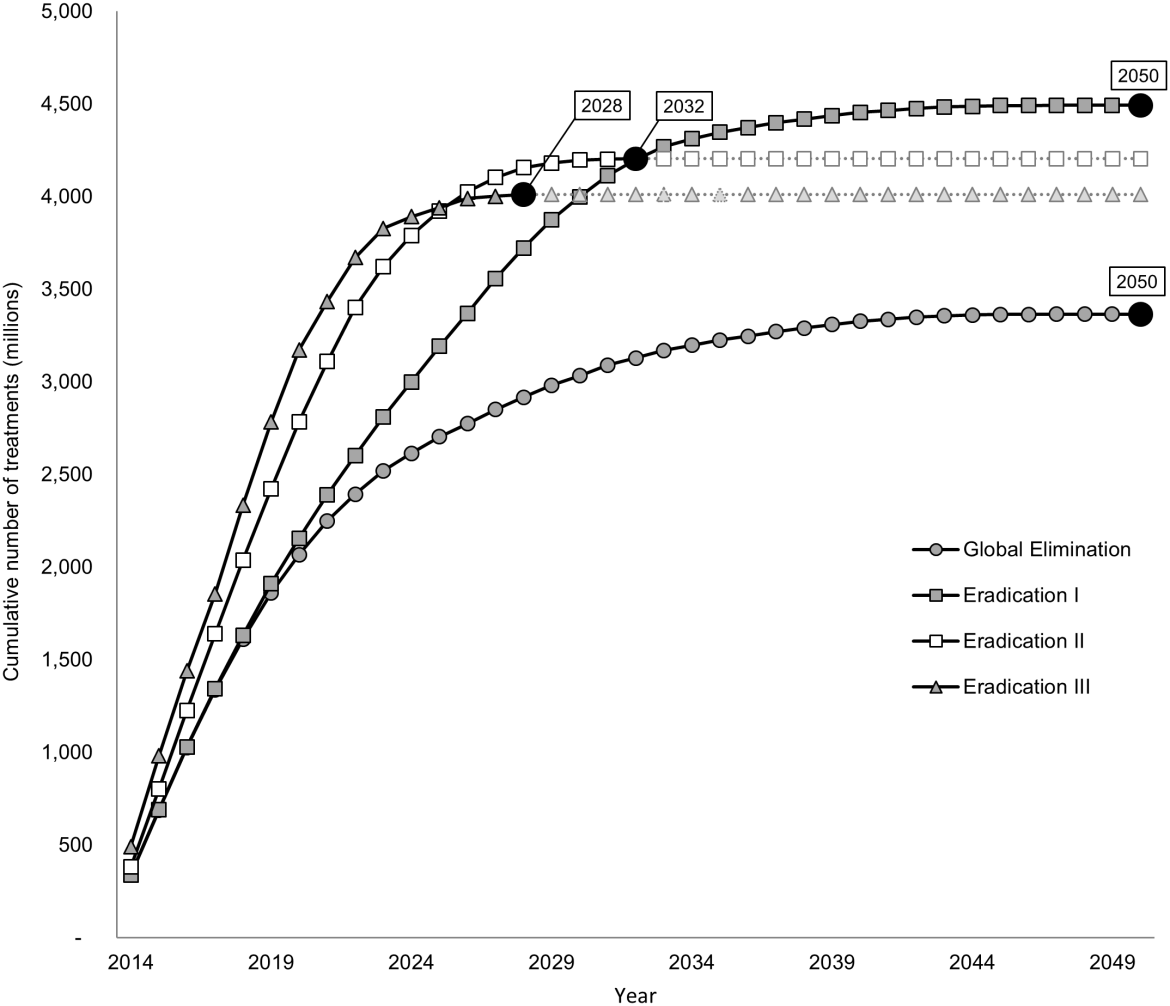
Cumulative number of treatments by year. The line with circular markers represents the global elimination (comparator) scenario. As highlighted in the text boxes, both the global elimination and eradication I scenario are estimated to conclude MDA after 37 years of MDA. Eradication II, the intensified scale-up scenario, sees the last round of MDA to occur by 2032, after 19 years of MDA. Eradication III is estimated to require 15 years of MDA, concluding in 2028.

**Fig 2:**
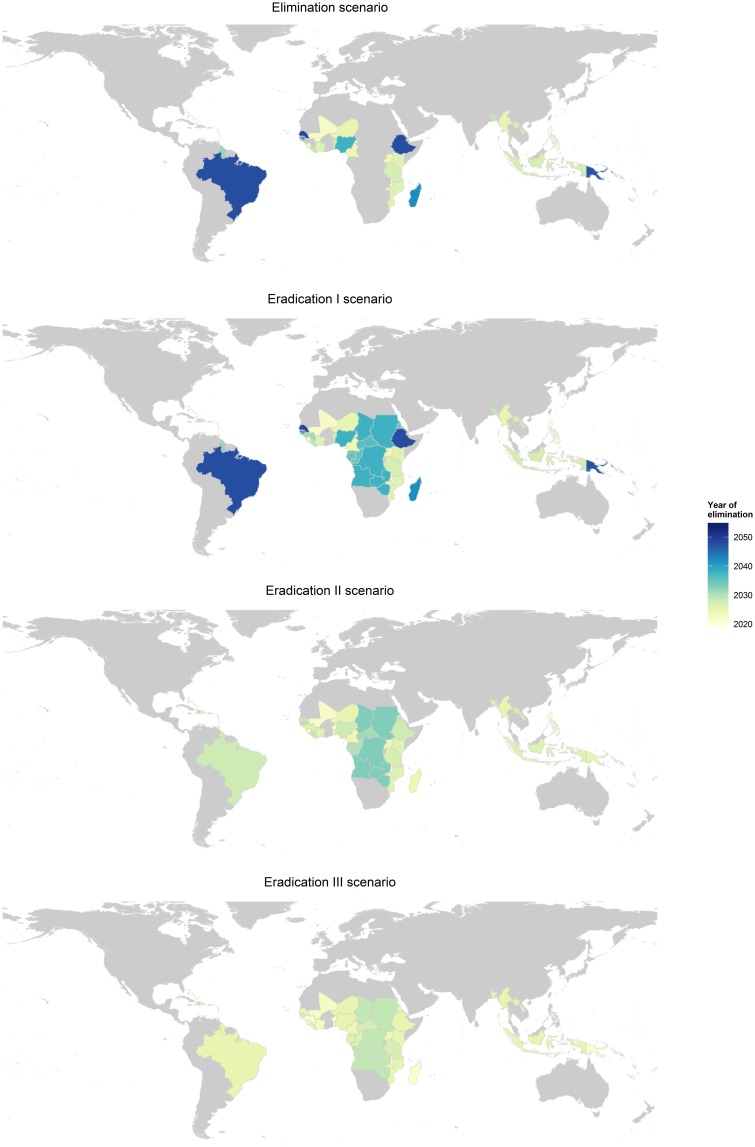
Maps depicting the final year of MDA per country for the four scenarios. The global elimination scenario does not include countries that have not yet begun MDA.

Our results indicate that interrupting LF transmission in all countries by 2020 is unlikely, though if MDA is drastically scaled-up and expanded, the final round of MDA to eradicate LF could be carried out by 2028 (eradication III; Fig 1). If scale-up continues at the current rate, as modeled in our global elimination and eradication I scenarios, the last round of MDA will not be given until 2050, largely due to slow scale-up in areas where transmission occurs through *Culex* spp. The eradication II scenario reaches the last round of MDA by 2032. As this scenario assumes that all countries add 20% of their at-risk populations to MDA annually, the last countries to reach local elimination are those that were delayed due to mapping, and whose vector and treatment combination included *Anopheles* spp. and ivermectin or *Culex* spp. and DEC, including: Angola, Chad, the Democratic Republic of Congo, South Sudan, Sudan, Zambia, and Zimbabwe. Fig 2 provides a visual representation of the impact different intensities of scale-up and expansion have on time to reach local elimination for each country.

**Fig 3:**
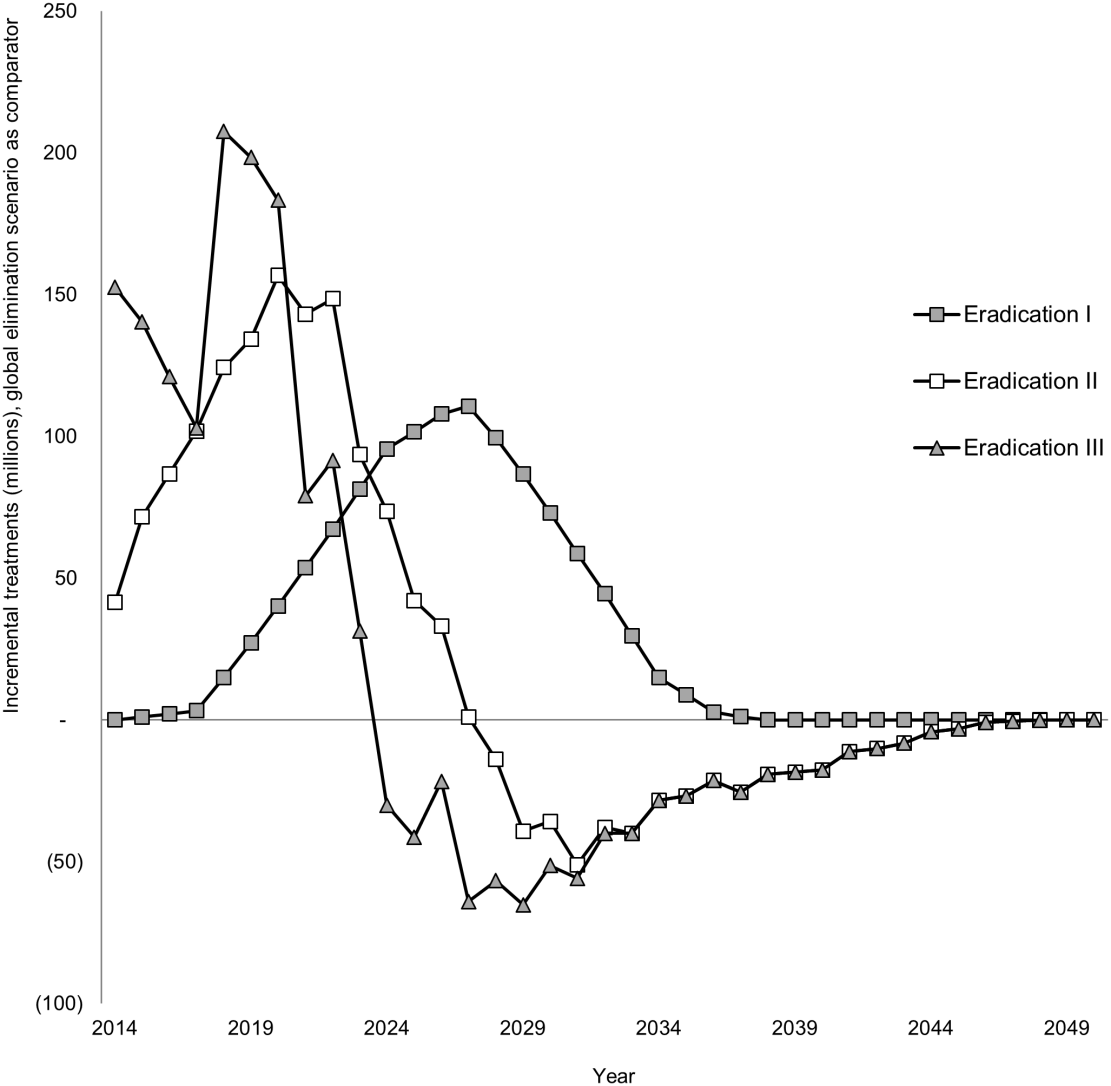
Incremental treatment projections by year (global elimination scenario as comparator). All eradication scenarios see an increase in the number of treatments after 4 years as the result of the imposed delay for countries that have not previously finished mapping or begun MDA. By 2024, the eradication III scenario requires less treatments than the global elimination (comparator) scenario, and from 2028, the eradication II scenario is also projected to require fewer treatments than global elimination.

Since the scenarios take into account population growth, rapid scale-up of MDA also decreases the number of treatments required. As depicted in Fig 3, the eradication III scenario initially requires substantially more treatments, but by 2024, the treatments under this scenario are projected to be less than that required under all other scenarios. The global elimination scenario is projected to require approximately 3,409 million treatments (95% CI: 3,185m–3,538 million). Expanding the program to all endemic countries will increase the number of treatments to 4,666 million (95% CI: 4,419m–4,904 million). Scaling up MDA more rapidly, as under the eradication II scenario, results in savings of nearly 300 million treatments compared to the eradication I scenario. Under the most optimistic scenario (eradication III), eradication could be achieved with 4,159 million treatments (95% CI: 3,924m–4,382 million). As shown in Fig 1, this represents nearly 750 million treatments more than the global elimination scenario but 210 million treatments less than the intensified eradication scenario (eradication II). Owing to the largest burden, the AFRO region requires the majority of treatments, followed by Southeast Asia. With the shift from global elimination to eradication, the number of treatments required in the Eastern Mediterranean region increases by more than 380 fold due to treatments required for Sudan, which is not considered under the elimination scenario (Table 5).

**Table 5:** Projected treatment needs (in millions) by WHO region with 95% credible intervals.

	Global elimination (comparator)	Eradication I	Eradication II	Eradication III
**AFRO**	2,117 (2,011–2,223)	3,202 (3,048–3,355)	2,930 (2,788–3,074)	2,746 (2,605–2,889)
**SEAR**	1,148 (1,102–1,190)	1,148 (1,102–1,190)	1,141 (1,096–1,183)	1,139 (1,096–1,181)
**WPR**	109.3 (104.5–114.0)	109.7 (104.9–114.4)	100.1 (95.6–104.7)	98.55 (94.25–102.94)
**AMR**	34.66 (33.07–36.27)	34.66 (33.07–36.27)	33.43 (31.87–35.00)	33.10 (31.60–34.62)
**EMR**	0.3729 (0.3380–0.4095)	173.0 (165.2–180.9)	164.1 (156.6–171.5)	142.0 (134.2–150.2)
**Total**	3,409 (3,185–3,538)	4,667 (4,419–4,904)	4,369 (4,133–4,594)	4,159 (3,924–4,382)

## Discussion

As not all LF endemic countries are considered under the global elimination (comparator) scenario, any eradication campaign will require a massive increase in treatments. However, if LF is to be eliminated in all endemic countries, then rapid scale-up as soon as possible will lead to increased savings—both in terms of time and treatments. Accelerated MDA may also propel the program towards success, as the risk of failure (due to lapses in funding, donor fatigue, or occurrence of calamitous events) potentially increases with extended program duration [33]. It is conceivable that a decrease in program duration may also decrease the likelihood of drug resistance evolution [34].

Noticeably missing from our analysis is India. While India has the greatest burden of LF [35], it has made substantial progress against the disease, having distributed nearly 3.5 billion antifilarial treatments since 2001 [13]. As such, our model suggests that further rounds may not be necessary for India. However, previous studies have found pockets of systematic non-compliance in India, leading to MDA coverage in those areas to fall below effective coverage [36]. It is therefore possible that transmission of LF may still occur in India. However, in order to remain consistent in our approach, and in recognizing that to provide global estimates we cannot take into account all eventualities, additional treatments for India have not been considered.

We sought data from a number of diverse sources. Due to the inherent structure of the LF program, however, our analysis relies heavily on data that have been collected and reported directly by each country. While this arrangement raises a number of issues, discrepancies in the data could also decrease the validity of our estimates. Inconsistencies in coverage data may affect the number of years required to interrupt transmission, while inaccuracies in at-risk estimations would directly impact the number of treatments projected to reach our scenario endpoints. Whether these issues would result in underestimates or overestimates is dependent upon the direction and magnitude of the error.

While we avoided underestimating scale-up potential through our eradication III scenario, it is possible that we overestimated the capacity of some countries to scale-up. It is possible that we also overestimated the effectiveness and ability to proceed with rapid scale-up in areas co-endemic with *L*. *loa*. While WHO has provisional guidelines for dealing with LF and *L*. *loa* co-endemicity, no such areas have been broadly targeted for LF elimination as yet, and thus the effectiveness and feasibility of the strategy remains unclear. At the same time, the mass distribution of long-lasting insecticidal nets (LLINs) in many malaria endemic sites is likely to have a large impact on LF transmission by anophelines [37, 38]. Because the impact remains difficult to quantify, and uncertainty remains regarding the duration LLINs have to remain in place, we have not included this here. The time and treatment estimates in this study are based on data and model formulations and parameterizations currently available to the authors. Many of the assumptions and simplifications inherent to our scenarios are in need of closer investigation. Ideally, models would be fit to specific transmission settings within and between countries, as parameter values have been shown to differ by region [29]. Other aspects equally deserving of more attention, but likewise beyond the scope of this project, are the effectiveness of twice-yearly albendazole in concert with vector control for areas co-endemic with *L*. *loa*, and the consequences of mid-program delays, [39, 40]. Care should thus be taken when interpreting these results, particularly at a country-specific level.

Our duration estimates are considerably longer than those proposed under the GPELF, which envisages all endemic countries to reach full geographic coverage by 2016, with post-MDA surveillance in all countries anticipated by 2020 [17]. While this level of scale-up is similar to that proposed under our eradication III scenario, we project the last round of MDA to occur nearly a decade later, in 2028. This divergence arises from differences in the assumed number of rounds of MDA required to interrupt transmission. Depending on baseline prevalence and vector-treatment combinations, our model estimates interruption in transmission to occur after 6–15 rounds of MDA (Table 4). In contrast, the GPELF assumes five years of MDA in all areas [17]. It is worth noting that the durations in this study represent a potentially conservative measure, as they were based on the 95^th^ percentile range of simulations leading to elimination, accounting for the uncertainty in our parameter estimates. This measure was taken to represent the time that could guarantee elimination with a reasonable level of certainty, but does not preclude that shorter durations may be sufficient in many areas. However, the discrepancy between predicted MDA durations and those advocated by GPELF was also evident in previous estimates with both deterministic and stochastic LF transmission models [41]. While aggressive goals for disease elimination and eradication potentially propel campaigns forward, overly optimistic projections could stifle innovations and further investment, ultimately hindering the initiative.

This study adds to the growing body of evidence on the feasibility of eradicating LF. While our estimates suggest more time may be needed to reach LF elimination than what is currently projected, the treatment estimates for our scenarios represent 66–89% of that which has already been distributed under the GPELF. Thus, our analysis indicates that with continued commitment, eradicating LF is within reach.

## Supporting Information

S1 File Supplementary text I: LF model description. (DOCX)

S1 Fig Example of microfilariae prevalence levels associated with the set of posterior estimates for anopheline transmission (10% prevalence). (TIF)

S2 Fig Examples of parameter value estimates for different vector genera and MF prevalence levels. (TIF)

S3 Fig Median values (solid lines) and 95^th^ percentile range (shaded areas) of LF prevalence for LF transmission by *Anopheles* spp. (left) and *Culex* spp. (right) at four different stable levels of pre-intervention LF prevalence. From top to bottom: 5, 10, 15, 20%, using diethylcarbamazine citrate and albendazole (red) or ivermectin and albendazole (blue) combination therapy.
(TIF)

S1 Table Parameter descriptions and values used. (DOCX)
