# Supplementary material for: What Is Needed to Eradicate Lymphatic Filariasis? A Model-Based Assessment on the Impact of Scaling Up Mass Drug Administration Programs
Source: PLoS Negl Trop Dis. 2015 Oct 9;9(10):e0004147. doi: 10.1371/journal.pntd.0004147 (PMC4599939; doi:10.1371/journal.pntd.0004147)
Supplement: S1 File — (DOCX) [file pntd.0004147.s001.docx]

**Supplementary text I: Lymphatic filariasis model description**

The deterministic transmission model used for the current analysis, EpiFil, has been described in detail, validated against multiple data sets for transmission settings with both *Anopheles* spp. and *Culex* spp. as vectors, and used extensively to predict outcomes of interventions (MDA, vector control) for Bancroftian lymphatic filariasis control [[1-4](#_ENREF_1)]. Specifically, the model versions we used largely followed the structure presented by Gambhir & Michael [[2](#_ENREF_2)], which includes the possibility of female worms remaining unmated in humans at low densities, and provides different microfilariae uptake functions (facilitation versus limitation) for *Anopheles* spp. and *Culex* spp. vectors.

The model consists of the following partial differential equations used to describe changes in state parameters:

$$\frac{\partial W}{\partial t}+\frac{\partial W}{\partial a}=\lambda\frac{V}{H}\psi_{1}\psi_{2}s_{2}h\left( a \right)L^{*}e^{-\beta I}-\mu W$$

$$\frac{\partial M}{\partial t}+\frac{\partial M}{\partial a}= \alpha\phi\left( W,k \right)W-\gamma M$$

$$\frac{\partial I}{\partial t}+\frac{\partial I}{\partial a}= W-\delta I$$

The state parameters represent the following: the mean adult worm burden in humans, W; the mean microfilariae density in humans, M; the mean level of immunity to infection, I. The initial conditions were W(0,t) = M(0,t) = I(0,t) = 0, while W(a,0), M(a,0) and I(a,0) were the equilibrium levels in the absence of interventions, obtained numerically by simulating the model for a sufficiently long period. The mean L3 density in the mosquito population, L*, is given by:

$$L^{*}=\frac{\lambda kg\int\pi(a)(1-f(M))da}{\sigma+\lambda\psi_{1}}$$

and f(M), which combines the moment generating function of the negative binomial distribution of infection in humans with the microfilariae uptake curve of individual mosquitoes, as:

$${f\left( M \right)}_{C}=(1+\frac{M}{k\left( M \right)}{(1-e^{-r/k}))}^{-k(M)}$$

$${f\left( M \right)}_{A}=\left[ \frac{2}{(1+\frac{M}{k\left( M \right)}{(1-e^{-r/k}))}^{k(M)}}-\frac{1}{(1+\frac{M}{k\left( M \right)}{(1-e^{-2r/k}))}^{k(M)}} \right]$$

with f(M)_C_ describing the function used for *Culex* spp. [[1](#_ENREF_1)] and f(M)_A_ for *Anopheles* spp. [[2](#_ENREF_2)]. The worm mating function is given by:

The rate parameters and values used are described in Table S1, while h(a) and π(a) represent the age-dependent attractiveness to mosquitoes (we assumed a linear increase from 0 to 1 over the first 10 years of life and a value of 1 for further years) and an approximation of the human age distribution (π(a) = 0.035 e^-0.026 a^), respectively, as in Norman et al [[1](#_ENREF_1)]. Typical values are presented as examples and for *Culex* spp. followed that of Norman et al [[1](#_ENREF_1)], who fitted parameter values to reflect transmission of *Wuchereria bancrofti* by *Culex quinquefasciatus* in an Indian environment. For *Anopheles* spp. the typical parameter values reflect the average of those presented by Gambhir *et al.* [[3](#_ENREF_3)] for the Tanzanian sites Tawalani and Masaika. However, as different geographic settings can differ dramatically in their parameter estimates [[3](#_ENREF_3), [4](#_ENREF_4)], and because we had a need for varying parameter estimates that would result in stable prevalence levels associated with our transmission archetypes (ca. 5%, 10%, 15%, and 20% prevalence), we obtained parameter sets that would lead to these levels of prevalence while allowing for parameter uncertainty.

In order to do so, we used a Bayesian framework of importance resampling [[3](#_ENREF_3), [5](#_ENREF_5)]. We first defined uninformative ranges for the parameter values based on literature and intuition (Table S1), and drew 10,000 sets of random samples from these uniform priors. For each of these randomly generated parameter sets, *i*, the model was simulated for 250 years at which point the stable equilibrium prevalence, *x*, was calculated. The goodness-of-fit of each run to the prevalence level associated with the transmission archetype, *p*, was estimated as a binomial likelihood, . We then randomly sampled, with replacement, 500 parameter sets from the original 10000 sets proportional to their likelihood, to obtain an approximation of a posterior distribution.

***Figure S1*: Example of microfilariae prevalence levels associated with the set of posterior estimates for anopheline transmission (10% prevalence).**

***Figure S2*: Examples of parameter value estimates for different vector genera and prevalence levels.**

We used these resampled parameter sets to investigate the impact of MDA on LF prevalence over time. Examples of prevalences associated with the parameter sets and distributions of a number of parameters are given (Figures. S1, S2).

We simulated the effects of filaricidal treatment by including a once-yearly instantaneous killing of a proportion of adult worms, μ_w_, and microfilariae, μ_mf_, depending on drug type and level of coverage. Additionally, fecundity of worms, 𝜶, was reduced for six to nine months following an MDA round by a proportion, μ_α_. The impact of MDA programmes was then investigated by repeating these treatments for a varying number of years, after which no further intervention took place. For each duration (number of MDA rounds), per vector type and drug regimen, we ran 500 simulations drawing from the range of posterior parameter estimates, and the lowest number of rounds at which in the 95^th^ percentile range of these simulations prevalence was below 1% and decreasing at the end of the simulation was taken as a conservative measure of the number of rounds required to ensure elimination. Examples of simulations leading to interruption of transmission are given in Figure S3, and the predicted number of rounds required are provided in Table 3.

**Figure S3: Median values (solid lines) and 95^th^ percentile range (shaded areas) of LF prevalence for LF transmission by Anopheles spp. (left) and Culex spp. (right) at 4 different stable levels of pre-intervention LF prevalence**. From top to bottom: 5, 10, 15, 20%), using DEC and albendazole (red) or ivermectin and albendazole (blue) combination therapy.

**References cited in supplementary text:**

1. Norman, R., et al., *The development of an age-structured model for describing the transmission dynamics and control of lymphatic filariasis.* Epidemiology and Infection, 2000. **124**: p. 529-541.

2. Gambhir, M. and E. Michael, *Complex ecological dynamics and eradicibility of the vector borne macroparasitic disease, lymphatic filariasis.* PLoS ONE, 2008. **3**: p. e2874.

3. Gambhir, M., et al., *Geographic and ecologic heterogeneity in elimination thresholds for the major vector-borne helminthic disease, lymphatic filariasis.* BMC Biology, 2010. **8**: p. 22.

4. Singh, B., et al., *Sequential modelling of the effects of mass drug treatments on anopheline-mediated lymphatic filariasis infection in Papua New Guinea.* PLoS ONE, 2013. **8**: p. e67004.

5. Smith, A. and A. Gelfand, *Bayesian statistics without tears: a sampling-resampling perspective.* The American Statistician, 1992. **46**: p. 84-88.
