# Supplementary figures and images for: What Is Needed to Eradicate Lymphatic Filariasis? A Model-Based Assessment on the Impact of Scaling Up Mass Drug Administration Programs
Source: PLoS Negl Trop Dis. 2015 Oct 9;9(10):e0004147. doi: 10.1371/journal.pntd.0004147 (PMC4599939; doi:10.1371/journal.pntd.0004147)

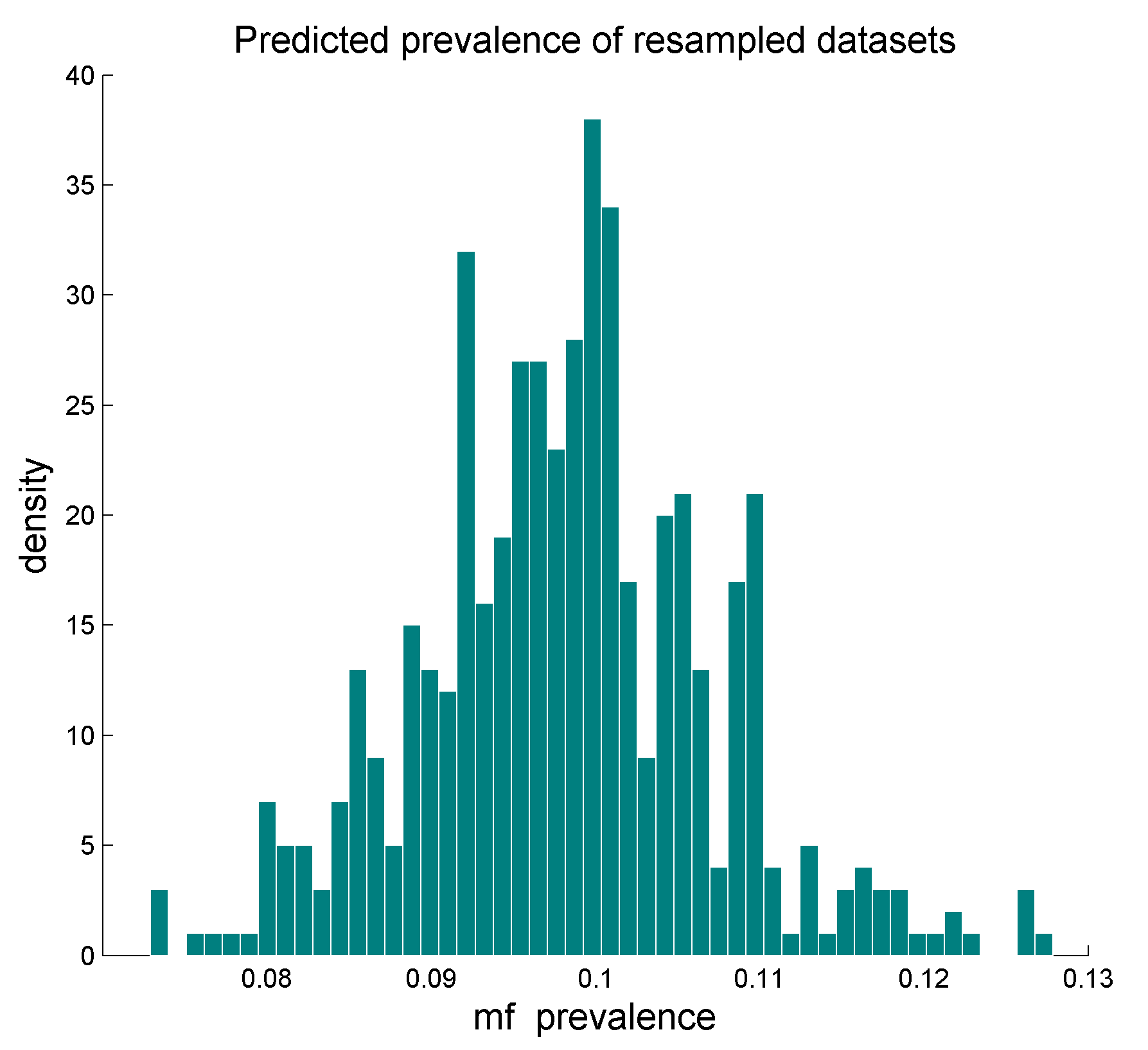

Supplement: S1 Fig — (TIF) [file pntd.0004147.s002.tif]

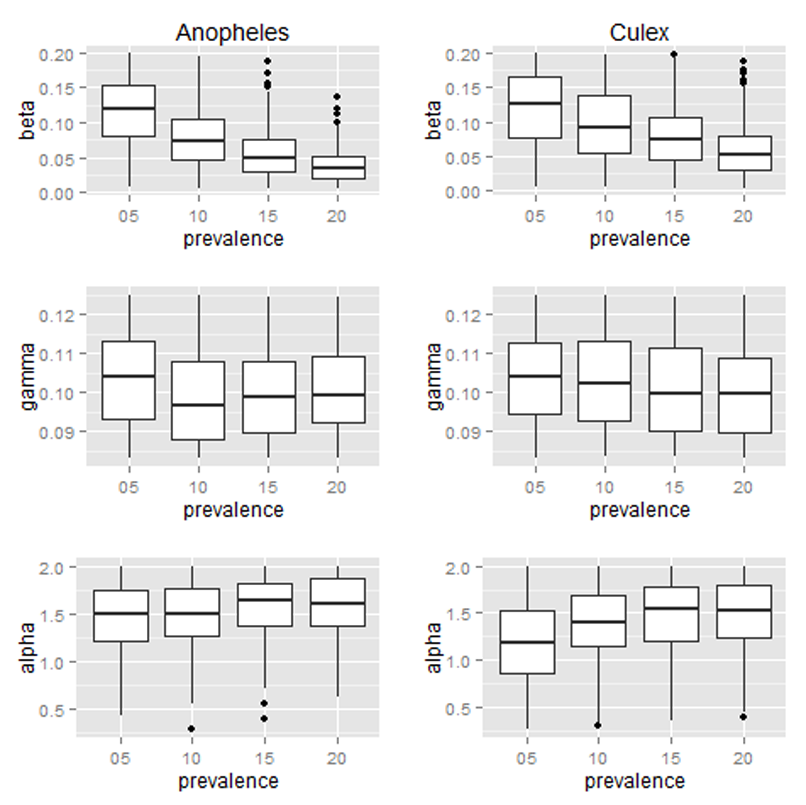

Supplement: S2 Fig — (TIF) [file pntd.0004147.s003.tif]

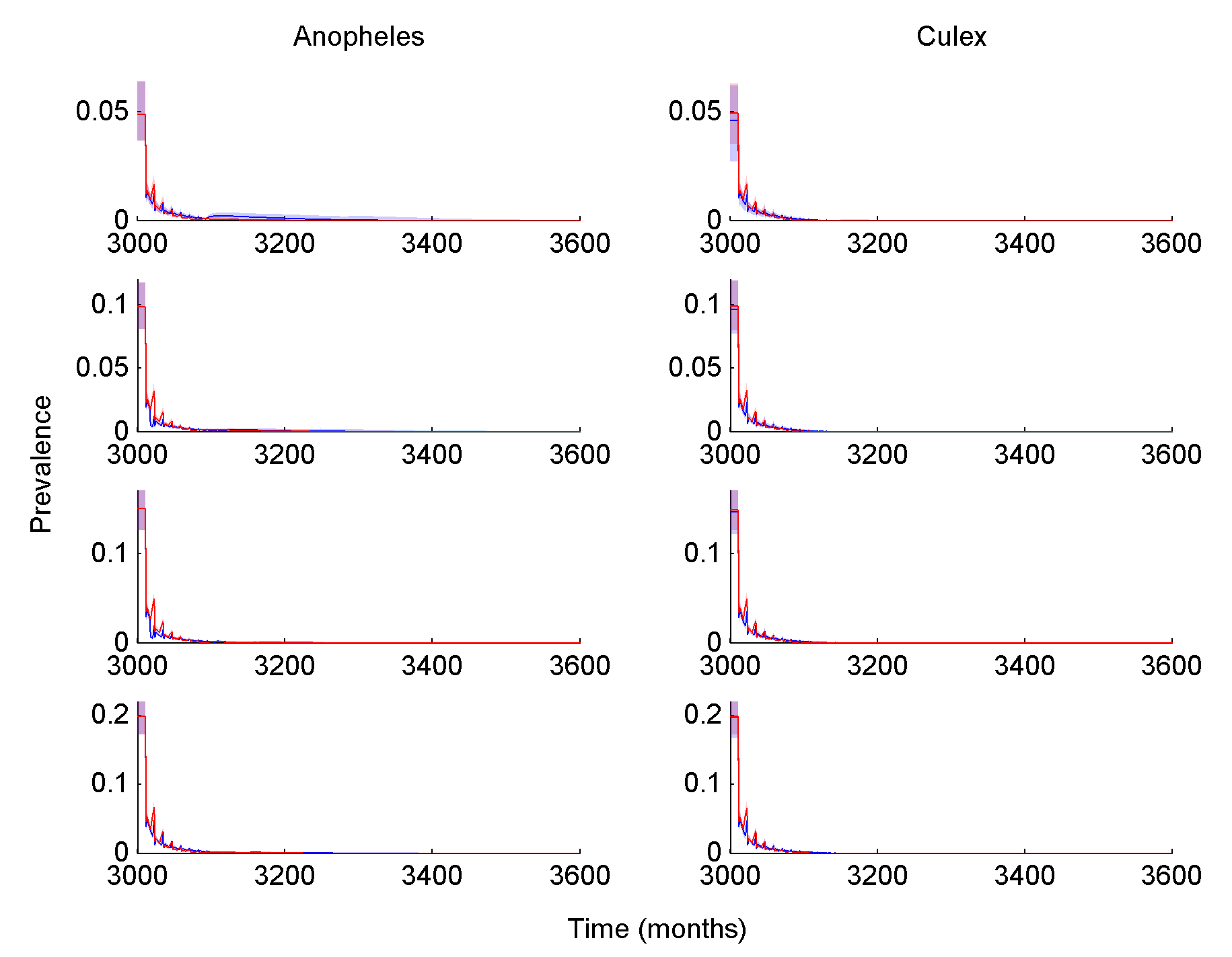

Supplement: S3 Fig — From top to bottom: 5, 10, 15, 20%, using diethylcarbamazine citrate and albendazole (red) or ivermectin and albendazole (blue) combination therapy. (TIF) [file pntd.0004147.s004.tif]
