# Supplementary material for: What Is Needed to Eradicate Lymphatic Filariasis? A Model-Based Assessment on the Impact of Scaling Up Mass Drug Administration Programs
Source: PLoS Negl Trop Dis. 2015 Oct 9;9(10):e0004147. doi: 10.1371/journal.pntd.0004147 (PMC4599939; doi:10.1371/journal.pntd.0004147)
Supplement: S1 Table — (DOCX) [file pntd.0004147.s005.docx]

**Table S1: Parameter descriptions and values used**

| *Parameter* | *Description* | *Typical value* | |  |
| --- | --- | --- | --- | --- |
|  |  | *Culex^†^* | *Anopheles^‡^* | *Prior ranges* |
| λ | human biting rate | 10 | 10.52 | 6-15 (0.2-0.5 per day) |
| V/H | vectors per human | 576 | 75.5 | 50-500 |
| ψ_1_ | proportion of L3s leaving per bite | 0.4 | 0.45 | 0.3-0.7 |
| ψ_2_s_2_ | proportion of L3s entering puncture & establishing | 0.0001 | 0.004 | 0.00002-0.004 |
| g | proportion of bites on infected humans leading to infection | 0.37 | 0.37 | 0.25-0.5 |
| β | measure of acquired immunity | [0.031 - 0.11] | [0.011 - 0.047]*^#^* | 0.001-0.2 |
| δ | decay of immunity | 0.004 | 0 | 0-0.005 |
| μ | adult worm death rate | 0.01 | 0.01 | 0.014-0.007 |
| 𝜶 | adult worm fecundity | 2 | 1.14 | 0.2-2 |
| σ | mosquito death rate | 5 | 4.68 | 0.9-6 |
| γ | death rate of mf | 0.1 | 0.095 | 0.083-0.125 |
| C | MDA coverage | 85% | 85% | - |
| μ_mf_ | microfilaricidal effect | 0.95; 0.99 | 0.95; 0.99 | 0.9-0.95; 0.95-0.99 |
| μ_W_ | macrofilaricidal effect | 0.55; 0.35 | 0.55; 0.35 | 0.5-0.6; 0.3-0.4 |
| μ_𝜶_ | suppression of worm fecundity | 0.95; 0.99 | 0.95; 0.99 | 0.9-0.95; 0.95-0.99 |
| κ | constant in L3 uptake function | 6 | 4.39 | 4-6 |
| r | constant in L3 uptake function | 0.047 | 0.055 | 0.04-0.06 |
| k(M) | aggregation parameter | 0.0029 + 0.0236(M) | 0.00203 + 0.015(M) | (0.0006-0.002) + (0.01-0.03) M |

*^†^*Parameter values for *Culex* taken from Norman et al (2000)

*^‡^*Parameter values for *Anopheles* taken from Gambhir & Michael (2008) and Gambhir et al (2010)
